# A Green Nanostructured Pesticide to Control Tomato Bacterial Speck Disease

**DOI:** 10.3390/nano11071852

**Published:** 2021-07-18

**Authors:** Daniele Schiavi, Rosa Balbi, Stefano Giovagnoli, Emidio Camaioni, Ermelinda Botticella, Francesco Sestili, Giorgio Mariano Balestra

**Affiliations:** 1 Department of Agricultural and Forestry Sciences (DAFNE), University of Tuscia, Via San Camillo de Lellis snc, 01100 Viterbo, Italy; ermelinda.botticella@ispa.cnr.it (E.B.); francescosestili@unitus.it (F.S.); 2 Department of Pharmaceutical Sciences (DSF), University of Perugia, Via del Liceo 1, 06123 Perugia, Italy; rosa.balbi@hotmail.it (R.B.); stefano.giovagnoli@unipg.it (S.G.); emidio.camaioni@unipg.it (E.C.); 3 CNR-Institute of Sciences of Food Production (ISPA), Unit of Lecce, Via Provinciale Lecce-Monteroni, 73100 Lecce, Italy

**Keywords:** cellulose nanocrystals, chitosan hydrochloride, starch, *pseudomonas syringae* pv. *tomato*, tomato, nanotechnology, microparticles, spray-drying, organic plant protection

## Abstract

Bacterial speck disease, caused by *Pseudomonas syringae* pv. *tomato* (Pst), is one of the most pervasive biological adversities in tomato cultivation, in both industrial and in table varieties. In this work synthesis, biochemical and antibacterial properties of a novel organic nanostructured pesticide composed of chitosan hydrochloride (CH) as active ingredient, cellulose nanocrystals (CNC) as nanocarriers and starch as excipient were evaluated. In order to study the possibility of delivering CH, the effects of two different types of starches, extracted from a high amylose bread wheat (high amylose starch—HA Starch) and from a control genotype (standard starch—St Starch), were investigated. Nanostructured microparticles (NMP) were obtained through the spray-drying technique, revealing a CH loading capacity proximal to 50%, with a CH release of 30% for CH-CNC-St Starch NMP and 50% for CH-CNC-HA Starch NMP after 24 h. Both NMP were able to inhibit bacterial growth in vitro when used at 1% w/v. Moreover, no negative effects on vegetative growth were recorded when NMP were foliar applied on tomato plants. Proposed nanostructured pesticides showed the capability of diminishing Pst epiphytical survival during time, decreasing disease incidence and severity (from 45% to 49%), with results comparable to one of the most used cupric salt (hydroxide), pointing out the potential use of CH-CNC-Starch NMP as a sustainable and innovative ally in Pst control strategies.

## 1. Introduction

Recent advances in nanotechnology have shown the concrete possibility of minimizing agrochemicals used in agriculture, especially those ones related to plant protection strategies [1]. The greatest risks deriving from the abnormal application of traditional pesticides are manly linked to environmental pollution, due to the low effectiveness of compound delivery. Nanomaterials and nanofunctionalized carriers could provide a better coverage when applied on crops, enhancing antimicrobial properties of pesticides thanks to controlled release of active ingredients, and at the same time reducing the chemical dispersion in soil and waters [2,3]. Besides, today a more sustainable agriculture is strongly requested in those supply chains where the use of traditional pesticides such as cupric salts is discouraged. In the current scenario, the aim of the present work was to investigate the potential use of a novel green pesticide on one of the most widespread crop all over the world (tomato) and on one of its most commonly associated disease (bacterial speck). Bacterial speck is known for being able to infect both table and industrial varieties of tomato. This disease is provoked by the Gram negative bacteria *Pseudomonas syringae* pv. *tomato* (Pst), which is able to produce symptoms on fruits, leaves and stems. Bacterial cells from the epiphytic phase can penetrate living tissue through stomata or microlesions aroused by leaves pruning and harvesting operations, but also by environmental agents, such as wind, hail or rain, causing the appearance of chlorotic specks that necrotize quickly [4,5]. Necrotic tissues can merge together, leading to a loss of photosynthetic efficiency in green organs and the presence of scars, which compromises fruit’s marketability. Moreover, the bacteria can survive on seeds, representing a huge threat in every tomato cultivation phase, from nursery to field [6]. Although the experimental application of alternative compounds has shown the potential capability of counteracting bacterial speck, nowadays tomato protection strategies still rely on the use of copper as a preventive tool to avoid bacterial infection and evasion [7,8,9]. Copper and its derivatives are well-known active principles for their antimicrobial activity against bacteria and fungi, ease of use and affordability, and due to that they are some of the most used worldwide [10,11]. On the other hand, several scientific studies highlighted the side effects of overusing copper in agriculture, including a negative impact on soil and water microorganisms and the appearance of resistant strains [12,13,14,15]. Governments are enacting different legislative measures in order to diminish the amount of usable copper in agriculture: in Europe, the European Commission, with the application of the executive regulation 2018/1981 of 13 December 2018, cut the maximum limit of usable copper from 6 kg per hectare per year (maximum 30 kg per hectare in five years) to 4 kg per hectare per year (maximum 28 kg per hectare in seven years), renewing the approval of the active substances copper compounds as candidates for substitution. The need to find alternative plant protection compounds in order to support farmers in plant protection strategies is mandatory. Several studies have already demonstrated the potential application of natural derived substances, such as phenols and essential oils, for controlling tomato bacterial diseases. Quattrucci et al. (2013) positively tested in vitro and in vivo the antimicrobial effects of *Punica granatum* peel extracts on Pst [16]. Morin, naringenin and phloretin, which are plant flavonoids, affect different Pst strains’ virulence, reducing flagellin production and disturbing cell swimming and swarming motility [17]. Ethanolic extract of *Magnolia tamaulipana* was able to inhibit almost 90% of Pst growth at concentration of 125 ppm, while a strong in vitro antibacterial activity of essential oils extracted from different plants such as mint, thyme and eucalyptus was recorded on Pst, due to the presence of phenolic compounds [18,19]. Chitosan and its derivatives are some of the most studied antimicrobial compounds, the application of which in agriculture is already allowed as resistance inductors [20,21]. Due to its antifungal, antibacterial and antioxidant properties, together with its ability of boosting plants defenses and biological parameters, such as photosynthetic activity, seeds germination and vegetative growth, Chitosan is nowadays one of the most promising allies in sustainable plant protection strategies, from seed coating to post-harvest [22,23,24,25,26]. Moreover, the application of chitosan is being pursued due to its economic convenience, since it can be obtained from agroindustrial wastes, like seafood shells, which currently represent, unlike the aforementioned substances, a very affordable starting point in defining innovative crop protection compounds [27,28]. Several scientific publications highlight the promising use of chitosan in tomato fungal (Fusarium wilt) and bacterial (bacterial spot and wilt) diseases management, showing good effects on vegetative parameters of plants [29,30,31,32]. Mansilla et al. (2013) demonstrated antimicrobial properties of chitosan on Pst, suggesting that inhibition could depend on several chemical parameters (pH, presence of metal ions, concentration), while the mode of action could be related to cell membrane interaction [33]. Biological properties of different chitosan forms mainly depend on acetylation degree, molecular weight, viscosity and concentration. Among the different forms of chitosan, the hydrochloride one, which was used in this study, derives from the interaction of chitin with hydrochloric acid, bestowing a greater hydrophilicity [34,35,36]. The issue with an effective use of chitosan and other natural molecules on plants is linked to their tricky miscibility, chemical instability and hard-to-control release. In this way, nanotechnology could represent a great breakthrough in sustainable pesticide formulation [2,37,38]. Antimicrobial and resistance inductance properties of several nanoparticles synthetized from inorganic materials, such as zinc, copper, magnesium, silver, silica and graphene, were already positively assayed against tomato bacterial spot and speck diseases [39,40,41,42,43]. Furthermore, many materials can be engineered to obtain carriers at the nanoscale level, which can be functionalized with active principles. In this sense, a very effective low-cost solution is represented by organic polymers [44,45,46]. Among these, cellulose nanocrystals (CNC) are some of the most promising, due to their well-known biochemical properties, including high elasticity modulus, thermal stability, no cytotoxic effects and high presence of hydroxyls groups, which can be exploited for active principles linking [47,48,49,50]. Cortesi et al. (2017) showed how natural substances like gallic acid can be encapsulated in cellulose microparticles and effectively used against bacterial speck agents, while in other works antimicrobial properties on Pst were demonstrated in polymeric films derived from the combination of CNC with poly (lactic acid) [51,52]. Additionally, Fortunati et al. (2016) assessed the potential use of CNC as novel nanocarriers for smart delivery systems after proving their biocompatibility on tomato plants [53]. In order to grant controlled release features, starch was also considered as an additive. Starch and different starch-based nanostructures are well-known excipients, frequently used in the food and pharmaceutical industries [54]. Starch can facilitate the passage in solution of molecules by adsorbing water. This ability is influenced by the content of amylose (linear glucose polymer) and amylopectin (branched glucose polymer), which are normally present in a 1:3 ratio, as well as the variable architecture of the starch matrix. Starch with a high amylose content (HA Starch) has a better film forming ability and a higher gelling strength compared to standard starch (St Starch) [55,56]. Depending on origin and structure, HA Starch shows useful properties in terms of modulation of active substance release, and its crosslinked forms are common excipients (Contramid^®^) in oral formulations [57,58]. With the aim of improving human nutrition, research is currently starting to focus on obtaining high amylose content wheat genotypes [59].

In this work, we evaluated the possibility of fabricating a novel ternary delivery system by the spray-draying technique, using CNC as nanocarriers for chitosan hydrochloride (CH), with the addition of standard starch or high amylose starch as possible release modifiers. Obtained nanostructured microparticles (NMP) were characterized for their biochemical properties, including effects on bacterial and plant growth in comparison with copper. To the best of our knowledge, this is the first time that a fully organic nanostructured pesticide is produced for the specific management of tomato bacterial speck disease.

## 2. Materials and Methods

### 2.1. Materials

Chitosan hydrochloride (CAS No. 70694-72-3) was purchased at Wuhan Victory Excellence Pharmaceutical Co., Ltd. (Wuhan, China). Microcrystalline cellulose (CAS No. 9004-34-6) was purchased at Sigma-Aldrich, Inc. (Taufkirchen, Germany) Standard starch and high amylose starch were extracted from the cultivar Cadenza and Cadenza SBEIIa, respectively. This latter line was recently produced by Botticella et al. (2018) through the silencing of a key gene involved in starch biosynthesis, the starch brancing enzyme of class IIa (SBEIIa) [60]. All other reagents used in this work were supplied by Sigma-Aldrich, Inc. (Taufkirchen, Germany), and used without further modifications.

### 2.2. CNC Synthesis and Characterization

#### 2.2.1. CNC Synthesis

Cellulose nanocrystals (CNC) were synthetized by acid hydrolysis, as described by Cranston and Gray (2006) [61]. Microcrystalline cellulose was briefly hydrolyzed in a sulphuric acid solution (64% w/w) under continuous stirring at 45 °C for 30 min. The obtained suspension was put in dialysis tubes until the pH reached 7. After that, CNC aqueous suspension went through an ultrasonic treatment (700 W for eight minutes). CNC were conserved at 4 °C.

#### 2.2.2. CNC Morphological and Chemical Characterization

Acid hydrolysis yield was calculated using the method reported as UNI EN ISO 638:2009. 

CNC morphology was investigated by field-emission microscope Zeiss LEO 1525 equipped with a GEMINI column (Oberkochen, Germany). Samples for SEM analysis were prepared by depositing CNC suspensions onto an aluminum specimen stub covered with a double-sided adhesive disc. The sample was coated with a chromium layer (8 nm thickness) before imaging (Quorum Q150T ES East Grinstead, West Sussex, UK). 

Absorption attenuated total reflection (ATR-IR) spectra were recorded with the Infrared Spectrophotometer IRSpirit (Shimadzu, Japan) in the spectral range of 400–5000 cm^−1^. The analyses were performed directly on the powders with no sample processing. Background spectra were acquired on the empty cell.

### 2.3. Starch Extraction and Characterization

#### 2.3.1. Starch Extraction

Starch granules were extracted from whole flour using the dough ball method as described in Botticella et al. (2018) [60]. Ten grams of whole flour were mixed with 0.6 parts (w/w) of water, and kneaded to form a dough. Starch was washed from the dough with 300 mL deionized water until the washing water was clear. After a centrifugation at 1500× *g*, the pellet was rinsed twice in 20 mL deionized water. The upper yellow-gray layer deposited on the surface of the starch pellet was manually removed with a spatula, and the starch left to dry for two days at room temperature.

#### 2.3.2. Starch Granule Morphology

The morphology of starch granules was analyzed using a scanning electron microscopy (SEM) Hitachi S-4000 (Krefeld, Germany), as reported by Sparla et al. (2014) [62]. 

#### 2.3.3. Quantification of Amylose, Total Starch and Resistant Starch

Amylose content was determined from 15 mg aliquot of purified starch through a colorimetric assay based on an iodine-amylose reaction [63]. A standard curve was generated from mixtures of potato amylose (Fluka, Neu-Ulm, Germany) and wheat amylopectin (Sigma Aldrich, St. Louis, MO, USA). Total starch and resistant starch were determined from whole flour samples, using the Total Starch (AA/AMG) test and Resistant Starch Assay kits (Megazyme, Irishtown, Ireland). For the determination of the total starch content, the protocol specified for “samples containing also resistant starch” was followed. 

For each analysis, three biological samples, each with three technical replicates, were measured either for Cadenza or Cadenza SBEIIa. 

### 2.4. Preliminary In Vitro Antibacterial Activity 

In order to define the most effective CH antibacterial concentration useful to assess the starting requirements for NMP preparation and in vivo application, as well as to investigate possible St Starch, HA Starch, and CNC antimicrobial effect, an in vitro assay was conducted.

#### Agar Incorporation Assay

Antimicrobial effects of substances were recorded using an agar incorporation method [64]. Pst (CFBP 1323) was grown on King’s B (KB) medium plates at 27 °C for 48 h [65]. Formed colonies were scraped off and suspended in deionized sterilized water to 10^8^ CFU mL^−1^. Concentration of bacterial suspension was adjusted until an OD600 = 1 was reached using a spectrophotometer [66]. A bacterial suspension of 104 CFU mL^−1^ was made. KB medium plates were amended with the different substances to reach 0.05, 0.1, 0.5 and 1% w/v concentrations. KB medium was used as negative control, while copper hydroxide at 0.09% w/v (field dose) was used alone as a positive control [67]. Plates have been allowed to cool, and then 100 μL of the bacterial suspension were gently plated on. After incubation at 27 °C for 48 h, formed colonies were counted using a stereomicroscope. Three replicates were made for each thesis. Minimal inhibitory concentration (MIC) of tested substances has been identified as the one where no visible colonies have grown, and so where inhibition percentage was equal to 100%. Inhibition of bacterial growth was expressed as a percentage, and calculated below:

Inhibition (%) = [(Neg. ctrl. abs.- Tested substance abs.) × Neg. ctrl. abs.^−1^] × 100 (1)

### 2.5. Synthesis and Charachterization of CH-CNC-Starch NMP

#### 2.5.1. Preparation and Characterization of CH-CNC-Starch NMP

An established amount of CNC was suspended in ultrapure water to obtain a concentration of 15 mg/mL. CH and St Starch or HA Starch were dissolved in the CNC suspension at 50:30:20 CH:CNC:Starch w/w ratio. The suspensions were then nebulized by a Büchi mini spray-dryer B-290 (Büchi^®^, Flawil, Switzerland) in the following conditions: inlet temperature 140 °C; aspirator rate 50%; feed rate 2.4 mL/min; and air pressure 357 L/h. The powders were recovered and stored at room temperature and 30% relative humidity until use. 

NMP morphology and IR spectroscopy analyses were performed on the powders according to the methods and conditions in paragraph 2.2.2.

In vitro release of CH from CH-CNC-Starch NMP was performed in PBS buffer 0.05 M pH 7 at 37 °C. The experiment was carried out in 50 mL falcon tubes. At established time points, an aliquot of 0.5 mL of PBS was collected and immediately replaced with the same volume of fresh PBS maintained at the same temperature. The amount of CH released was measured in triplicate by UV/Vis spectroscopy as reported above. The percent incremental release from the MP was plotted as a function of the incubation time. The experiment was run in triplicate, and data was reported as mean ± S.D. 

#### 2.5.2. CH Quantification Assay

CH standard solutions were prepared as follows. A CH solution was pipetted into test tube, and 0.1 mL of 0.5 M NaNO_2_ reagent was added. The mixture was then incubated at 80 °C for 30 min in a water bath to complete the depolymerization-deamination reaction. After depolymerization, the pH was raised to 8 by adding 0.2 mL NaOH (0.1 M), and the solution was then shaken. Subsequently, 1 mL of dinitrosalicylic acid reagent (DNSA, HiMedia), prepared as reported elsewhere [68], was added to the reaction mixtures, and the tubes were placed in a water bath at 75 °C for 15 min. The solution was briefly cooled, and the absorbance was measured at 540 nm by an Agilent 8453 diode array spectrophotometer (Agilent, Milan, Italy) against blank. A standard curve was built in the range 0.1–1 mg/mL (r^2^ = 0.999). 

#### 2.5.3. CH Extraction

The CH content (%CC w/w) in the CH-CNC-Starch NMP formulations was performed by dispersing an exactly weighed amount of NMP in 0.1 M acetic acid solution. The solution was incubated for 1 h at r.t. and periodically sonicated and vortexed, then treated according to the method for chitosan quantitation. All analyses were performed in triplicate, and the results expressed as mean ± standard deviation (SD).

#### 2.5.4. NMP In Vitro Antibacterial Activity

Antimicrobial effects of obtained NMP with different starches were recorded using a microdilution method [69,70]. A bacterial suspension of 10^6^ CFU mL^−1^ was made, after Pst was grown on KB medium at 27 °C for 48 h. Dilutions at 0.05, 0.1, 0.5 and 1% w/v were made for each NMP with LB broth. 198 μL of each dilution were pipetted in well of a 96 multiwell microplate. Then, 2 μL was of the bacterial suspension was added. LB broth was used as negative control, while copper hydroxide at field dose was used as positive control. For each thesis, a blank was made using only substances. Plates were incubated at 27 °C for 72 h. In order to study the possible bacteriostatic and bactericidal effects of NMP on Pst and their trend over time, at 48 and 72 h absorbance of plates at 600 nm was measured using a spectrophotometer. MIC has been identified as the one where inhibition percentage was equal to 100%. Inhibition of bacterial growth was expressed as a percentage, and calculated as below after subtracting the blank:

Inhibition (%) = [(Neg. ctrl. abs.-Tested substance abs.) × Neg. ctrl. abs.^−1^] × 100 (2)

where NMP shown a full inhibition of Pst growth, 100 μL from well were plated on a KB plate to assess the eventual bactericidal effects. Minimal bactericidal concentration (MBC) was defined as the one in which no colonies were recovered in plates after an incubation at 27 °C for 48 h. Eight replicates were made for each thesis.

### 2.6. CH-CNC-Starch NMP Phytobiological Compatibility

Obtained NMP were tested in vivo to assess their biocompatibility with living plant tissues. For each thesis, ten tomato seedlings of a local cultivar (San Marzano Scatolone) were picked up from an organic plant nursery, and have been acclimatized in a greenhouse with 75% air humidity, a 25/15 °C day/night thermoperiod and a 16/8 day/night photoperiod. Applied concentration of NMP was determined on results of antibacterial in vitro and quantification of loaded CH on NMP assays, as shown in the Result and Discussion paragraph. Two suspensions at 1% w/v were made with the two different NMP and sprayed manually all over the leaves and the stems, until a uniform coating was reached. Deionized sterilized water and copper hydroxide were used as controls. One, seven and fourteen days post-treatment (1, 7, 14 dpt), the following parameters were recorded as stated for each thesis: three random leaves per plant were picked up and their area was measured with the software ImageJ (version 1.51j8) (NIH, Bethesda, MD, USA) (accessed on Windows 10) (Microsoft, Redmond, WA, USA) [71]; three measurements were made for each plant on the second leaf to assess the chlorophyll and flavonols content, and afterwards the nitrogen balance index (NBI), by a non-destructive method, using a leafclip sensor (Dualex 4 Scientific, FORCE-A, Orsay Cedex, France) [67,72]. The experiment was repeated thrice.

### 2.7. In Vivo Antibacterial Activity

In order to investigate the effect of the synthetized NMP on the disease development in a plant, an in vivo assay was performed. Ten tomato seedlings for each thesis were collected and treated as described in paragraph 2.6. 24 h after treatments, a 10^6^ CFU mL^−1^ Pst water-suspension was obtained after the bacteria was grown on KB medium for 48 h at 27 °C, and homogeneously spray-inoculated on leaves and stems. Plastic bags were placed on plants one day before and after the inoculation to increase the air humidity and promote the stomata’s opening [67]. The experiment was repeated thrice.

#### 2.7.1. Bacterial Epiphytic Survival

The effect of the NMP on Pst epiphytic survival was monitored one, seven and fourteen days post-inoculation (1, 7, 14 dpi). Three random leaves per plants were collected and washed in a sterile plastic bag with a pH 7 phosphate buffer 0.05 M using a homogenizer (Stomacher 400 Circulator, Seward Ltd., Worthing, UK) set at 110 rpm for 30 s. For each sample, serial decimal dilutions were obtained, and a 100 μL aliquot was gently plated on sucrose nutritive agar dishes. Each dilution was plated three times and incubated a 27 °C for 48 h. After that, developed bacterial colonies were counted and divided for the tomato leaf area measured with ImageJ; results were expressed as CFU cm^−2^ [67].

#### 2.7.2. Disease Symptomatic Expression

Symptomatic expression of bacterial speck disease was monitored on leaves for the entire duration of the trial. Disease severity (DS) was calculated as the number of point-like necrosis per plant at 7 dpi. Proportional disease reduction per plant (DR) was also calculated as below:

Proportional disease reduction (%) = [(Neg. ctrl. DS-Tested substance DS) × Neg. ctrl. DS^−1^] × 100 (3)

Disease incidence (DI) was calculated for each thesis at 7 and 14 dpi as the percentage ratio between total symptomatic (necrotized) area and total area of the collected leaves for the assessment of Pst epiphytic survival [67]. 

### 2.8. Statistical Analysis

Collected data were analyzed using one-way analysis of variance (ANOVA). Statistical significance of means was studied with Tukey’s HSD *post hoc* test. *p*-values less than 0.05 were considered significant, while *p*-values of less than 0.01 were considered highly significant. Release data were analyzed by paired Student’s *t*-test at 95% significance level. 

## 3. Results and Discussion

### 3.1. CNC Synthesis and Characterization

**Figure 1:**
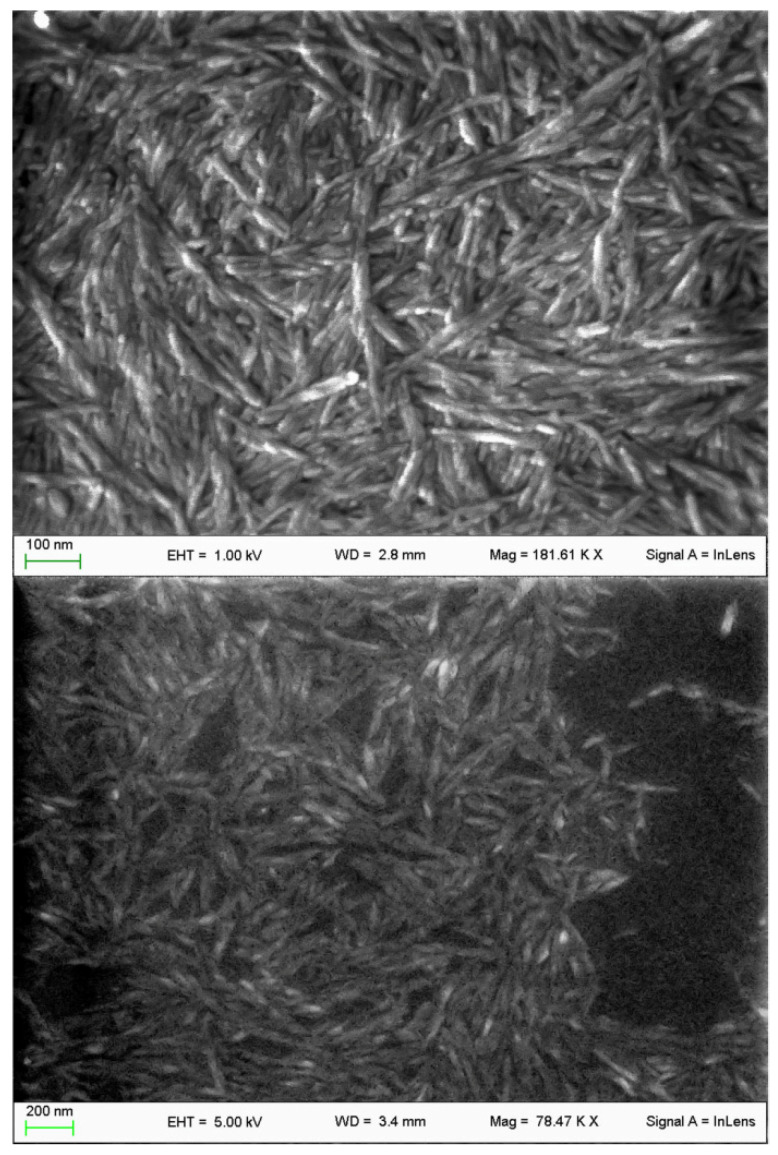
SEM microphotographs of the obtained CNC. Evident the typical elongated structure of nanocrystals and homogeneous size. Images were recorded at 78 (below) and 180 (above) kX.

CNC were successfully obtained through acid hydrolysis of microcrystalline cellulose with a total yield of about 20%. The method performance was consistent with relevant literature. SEM images show the typical needle shape of CNC with a certain homogeneity in size (Figure 1). The CNC IR spectral profile showed all the typical signals of CNC with no major changes compared to standard profiles. This excluded potential degradation effects of the harsh extraction conditions employed.

### 3.2. ST Extraction and Characterization

**Figure 2:**
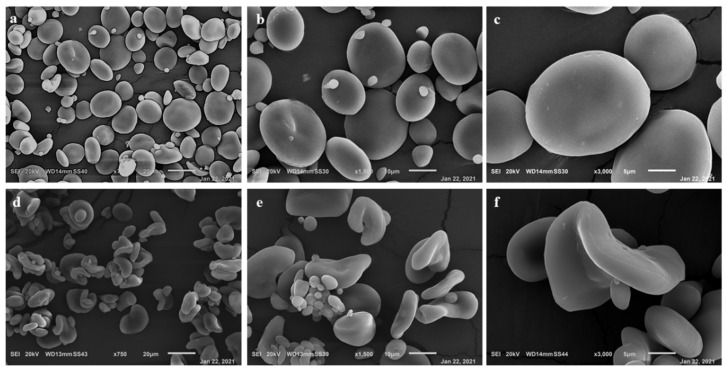
Scanning electron micrographs of isolated starch granules. (**a**–**c**) Cadenza (St Starch) and (**d**–**f**) Cadenza SBEIIa (HA Starch).

Morphological analysis of starch granules highlighted marked differences between Cadenza and Cadenza SBEIIa (Figure 2). In this latter genotype, type-A granules appeared deformed and deflated (Figure 2b) compared to the control (Figure 2a). Different type-B granules appeared more abundant, lost their normal spherical shape and became extended. Similar results were also observed by Botticella et al. (2018) [60].

No significant differences were observed for the total starch between Cadenza and Cadenza SBEIIa (Table 1). A different starch composition was determined between the control (Cadenza) and the high amylose genotype (Cadenza SBEIIa) (*p* < 0.05). In particular, the amylose contents were 32% and 63.1% of the total starch respectively (Table 1). A similar behavior was observed for the resistant starch; this fraction was higher in the high amylose genotype than the control (6.9% vs 0.8%).

### 3.3. Preliminary In Vitro Antibacterial Activity

**Figure 3:**
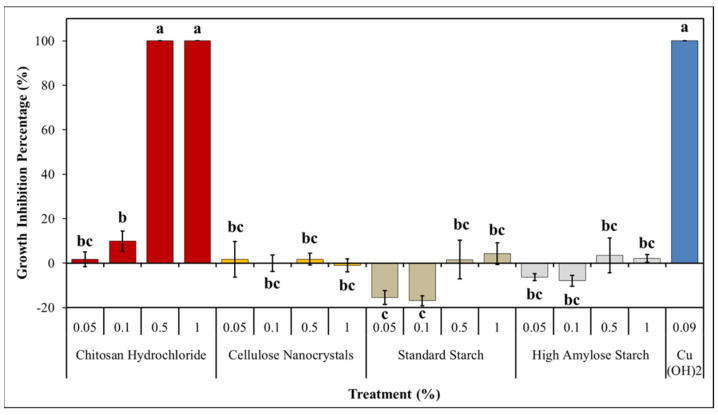
In vitro Pst growth inhibition percentage. MIC was assessed as the concentration on which inhibition on amended KB plates was equal to 100% after 48 h of incubation at 27 °C. Data are represented as the mean and SD, different letters (a, b, c) show significantly different values after one-way ANOVA, followed by Tukey’s HSD *post hoc* test were performed.

Pst growth inhibition was reported as a percentage (Figure 3). Experiment points out a statistically significant difference among these (*p* < 0.01). CH showed a total inhibition, comparable to copper hydroxide’s one, at both 0.5% and 1% w/v concentrations. MIC was established for chitosan hydrochloride at 0.5% w/v since lower tested concentrations have shown no significant activity on Pst growth. CNC showed no inhibition effects on Pst, and so both types of ST when used at 0.5% and 1% w/v concentrations. Lower concentrations of ST instead seem to promote Pst growth. This behavior is coherent with the ones showed in previous works, where chitosan, although in different forms, was able to explicate an antibacterial effect on several plant pathogenic bacteria [25,32,33]. Chitosan modes of action against bacteria still remain unclear, even if modern studies have suggested that they could be related to the cationic nature of NH_3_ groups present in chitosan molecules, which easily interact with negatively charged bacterial cell membrane. This interaction could lead to an alteration of membrane permeability, resulting in an inefficient capability of exchange molecules from outer to inner cell environment; Gram-negative bacteria, due to their higher presence of negative charges onto their membranes, appear to be more sensitive to chitosan [33,73,74]. Other mechanisms that were proposed to explain chitosan antibacterial activity are associated to the capability of disturbing biofilm formation, which is essential for disease development in some bacteria, and to the behavior of chelating metal ions, subtracting them to bacterial cells [75,76,77,78].

### 3.4. Synthesis and Charachterization of CH-CNC-Starch NMP

The obtained NMP showed good CH content very closed to the theoretical value of 50% w/w, as shown in Table 2. The powders had good homogeneity with %RSD values of 6.4 and 7.5. No major differences were observed when employing HA Starch or St Starch. A reasonable yield of the process slightly above 50% was achieved.

**Figure 4:**
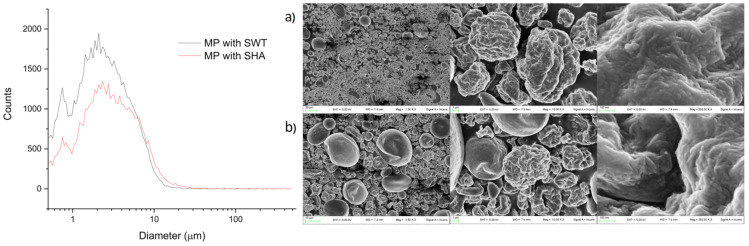
Particle size distributions (left) and SEM microphotographs (right) of the spray-dried NMP containing St Starch (**a**) or HA Starch (**b**). Picture were recorded at increasing magnifications. Images were recorded at 2.5, 10.0 and 250 kX, respectively.

The NMP showed overlapping size distributions with average size ranging around 2.9–3.8 µm (Figure 4). The analysis of particle morphology revealed at least two major dispersed populations: one formed by larger and smoother particles of about 10 µm in size and one constituted by smaller and rougher microparticles less than 5 µm in size (Figure 4a,b), which a closer observation confirmed to be formed by elongated nanostructures resembling those of CNC or starch material (data not shown). No relevant differences were observed when employing high amylose starch or standard starch.

**Figure 5:**
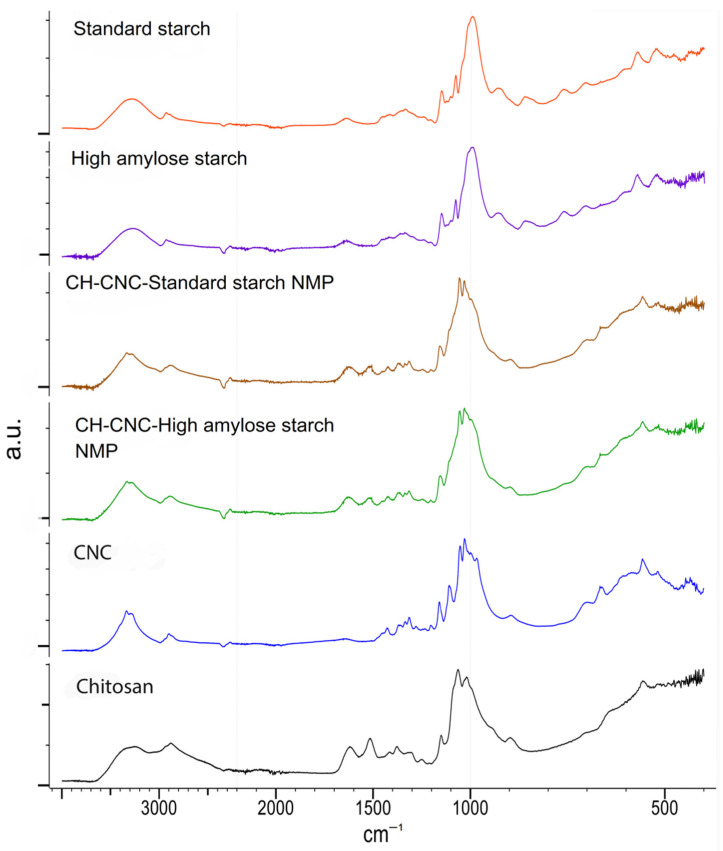
ATR-IR profiles of spray-dried NMP containing St Starch or HA Starch in comparison with the single formulation components. Spectra were acquired on unprocessed powders.

As already reported above for CNC, the IR spectra of CH, starches and CNC did not show particular changing in the main signals. For both NMP formulations, chitosan signals prevail over the other components with no significant differences. In particular, the amide bands of chitosan around 1640 and 1500 cm^−1^ are predominant in the NMP profiles regardless of the starch type employed (Figure 5). This is consistent with the CC values of nearly 50% for both NMP formulations, as reported in Table 2. 

On the other hand, most of the CNC and SHA or SWT signals overlap, with the only exception of a sharper CNC OH stretching around 3400 cm^−1^, which perhaps contributes to the corresponding band in the NMP profiles.

Overall, ATR-IR analysis showed that there is no major interaction among the different components, as no band shifts or distortions were observed.

**Figure 6:**
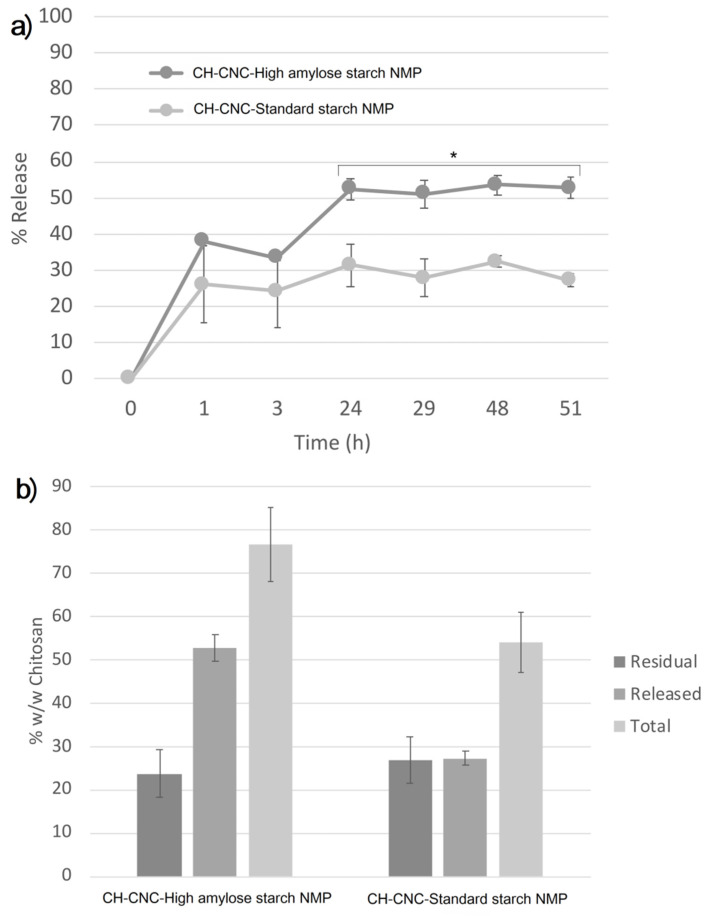
In vitro release profiles of chitosan from spray-dried NMP containing St Starch or HA Starch (**a**) and mass balance analysis (**b**) of the residual polymer remaining at the end of the time period investigated. Asterisk indicates significantly different values after one-way ANOVA, followed by Student’s *t*-test at 95% significance level were performed.

CH was initially released fast, with up to 40% of the polymer freed after 1h (Figure 6a).

The in vitro release profiles plateaued after 24 h, reaching around 50% for NMP with high amylose starch and 30% for NMP with standard starch. This behavior may be explained considering the gelling capacity of HA starch, which as observed in other cases [79], may enhance swelling thus promoting particle dispersion/dissolution and CH release.

However, mass balance analysis showed that the amount of CH remaining in the pellet at the end of the release period was about 30% w/w for both formulations. As a consequence, the total amount neared 80% w/w in the case of HA Starch and only 50% w/w for St starch, suggesting significant CH losses (Figure 6b). This behavior demands further investigation. Hypotheses include potential CH adhesion to surfaces, precipitation or other interactions with resulting detection issues and material loss. Further studies are also needed in order to investigate effects on release by modifying spray-drying parameters, such as components ratio, feed and aspirator rate, inlet temperature and air pressure. Although spray-drying isn’t the best performing technique in terms of regular size of obtained particles, spray-drying allows working with thermally sensitive compounds, such as natural substances.

**Figure 7:**
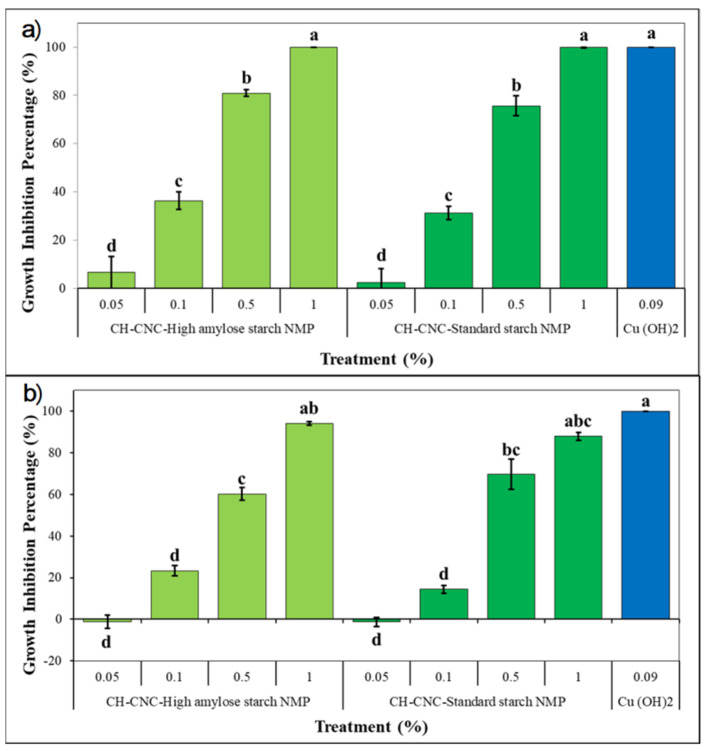
Pst growth inhibition of synthetized NMP was studied through a microdilution method. After 48 h (**a**) and 72 h (**b**) of incubation at 27 °C absorbance at 600 nm was measured for each sample. MIC was defined as the one in which inhibition was equal to 100%, while MBC was defined as the one in which no colonies formation was observed after plating bacterial suspensions. Data are represented as the mean and SD; different letters (a, b, c, d) show significantly different values after one-way ANOVA, followed by Tukey’s HSD *post hoc* test were performed.

In vitro antibacterial activity of both proposed NMP was tested using a microdilution method. Results were expressed as percentage as reported in Figure 7. At 48 h, a highly significant effect (*p* < 0.01) on Pst growth was recorded for CH-CNC-HA Starch NMP and CH-CNC-St Starch NMP when used at 1% w/v (Figure 7a). Similarly, to copper hydroxide treatment, a whole bacterial inhibition was reached. Results are consistent with those obtained in preliminary antibacterial assay, where MIC for CH was recorded at 0.5% w/v after 48 h. Indeed, CH content in NMP was seen to be equal to 50%. Surprisingly, an interesting inhibitory effect was recorded for both NMP when used at lower concentrations. In particular, growth inhibition values of 80.9 and 75.2% were noted at 0.5% w/v for CH-CNC-HA Starch NMP and CH-CNC-St Starch NMP, respectively, while when used at 0.1% w/v, NMP containing high amylose or standard starch showed values of 36.4 and 31.2%. The differences in antimicrobial activity of CH with respect to preliminary assay can be explained due the presence of starch, which was able to enhance CH dispersion. The functionalization of CH with starch is therefore a prosecutable way to boost antibacterial effectiveness while at the same time reducing the used amount of active principle. At 72 h, a similar behavior was recorded for both NMP with lower inhibition effects on Pst growth (Figure 7b). However, at 1% w/v concentration neither of two NMP was able to fully inhibit the bacterial growth, as their activity could be compared to copper hydroxide’s one (*p* < 0.01). Eventually, no bactericidal effect was recorded for tested substances, except for copper hydroxide, since Pst colonies formation was observed in plates after incubating the bacterial suspensions. The decrease in Pst inhibition during time and the absence of recorded values of MBC for tested NMP could find an explanation if CH antimicrobial effects are considered as bacteriostatic rather than bactericidal [73,78]. Obtained results induced the application of both NMP at 1% w/v in following in vivo experiments, in order to achieve the best antibacterial effect during time.

### 3.5. CH-CNC-Starch NMP Phytobiological Compatibility

**Figure 8:**
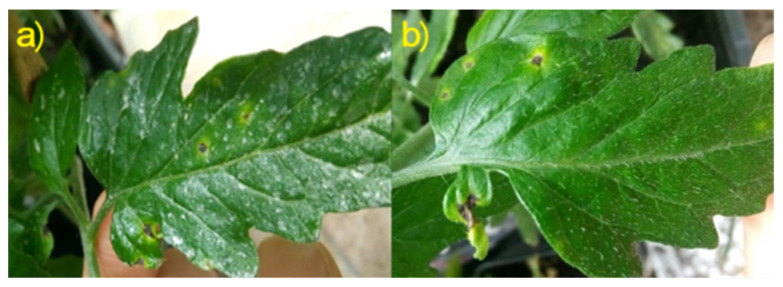
Particular tomato leaves treated with CH-CNC-HA Starch NMP (**a**) and with Water (**b**). Point-like necrosis surrounded by a chlorotic halo, which are the typical tomato speck symptoms, as well as the whitish patina that evenly covers the leaf when treated with NMP.

Biological parameters linked to plant basal physiological functions, such as leaf development and nitrogen use efficiency, were monitored in tomato seedlings in order to study the compatibility of tested NMP with plant growth, as reported in Table 3. At all the time points, no significant differences were recorded among the different theses in terms of developed leaf area. In the same way, no statistical differences were observed in leaf chlorophyll and flavonols content (Table 3). NBI, given by the ratio of these values, indicates the pathway of nitrogen in photosynthetic tissues, assuming that when plant status is healthy, most of its primary metabolism is involved in photosynthetic-related proteins synthesis, like chlorophylls ones, instead of taking part in flavonols pathway, which is associated to plant response to abiotic stresses [72]. Previous studies have demonstrated how CNC can be used without harmful effects on tomato plants, suggesting their potential use as nanocarriers [53]. Furthermore, chitosan effects on plant vegetative growth are positive when applied on leaves [20,26,31]. Our work strongly confirms this response, indicating in both formulation of CH-CNC-Starch NMP the capability of evenly covering tomato leaves without any detrimental side effects on basal vegetative parameters (Figure 8). The presence of a whitish spotty patina was noticed on leaves of plants treated with both NMP (Figure 8a)—its making should be due to the natural polymerization tendency of CNC and starch. Despite this, no phytotoxic effects were recorded on tomato seedlings during the trial, assessing the full compatible use of NMP on living tomato plants.

### 3.6. In Vivo Antibacterial Activity

**Figure 9:**
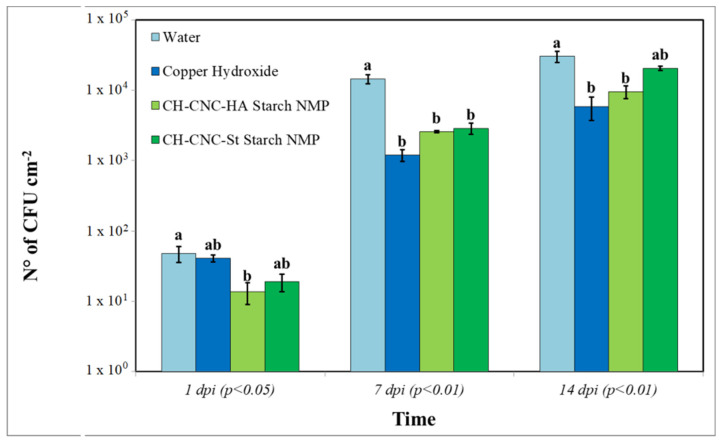
Pst epiphytical survival on tomato leaves at different time points. Three leaves per plant were collected, washed and measured in order to relate developed bacterial colonies to the leaf area. Data are represented as the mean and SD; different letters (a, b) show significantly different values after one-way ANOVA, followed by Tukey’s HSD *post hoc* test were performed.

Antibacterial effect of proposed NMP was assessed in vivo studying Pst epiphytic survival and symptomatic expression on artificially inoculated tomato leaves. At 1 dpi, CH-CNC-HA Starch NMP treatment showed a significant (*p* < 0.05) reduction in terms of bacterial epiphytic survival, with 1.37 × 10^1^ CFU cm^−2^. At 7 dpi, both tested NMP in a comparable way to the copper hydroxide, showed an highly significant (*p* < 0.01) effect on Pst epiphytic survival. N° of CFU cm^−2^ in plants treated with copper was equal to 1.20 × 10^3^, while for CH-CNC-HA Starch NMP and CH -CNC-St Starch NMP was 2.58 × 10^3^ and 2.88 × 10^3^, respectively. At 14 dpi, NMP with high amylose starch and copper hydroxide showed both a highly significant (*p* < 0.01) reduction of bacterial epiphytic survival recording values of 9.59 × 10^3^ and 5.84 × 10^3^ CFU cm^−2^ (Figure 9).

Symptoms monitoring on leaves during the trials has produced the following results: symptoms (chlorotic spots and necrosis) started to appear since the fifth day after inoculation. At 7 dpi, all plants belonging to different theses showed symptoms. DS calculated at 7 dpi showed a highly significant difference (*p* < 0.01) in developed point-like necrosis on tomato seedlings treated with both NMP with respect to the untreated sample; in particular, CH-CNC-HA Starch NMP has registered a mean value of 20.6 necrosis per plant, while CH-CNC- St Starch NMP has registered a mean value of 19.1 necrosis per plant. Necrosis count on plants treated with copper showed a reduction of 34.1%, while for NMP with high amylose starch and for NMP with standard starch values of 49.9% and 45.9% were reached (Table 4); nonetheless, these differences were not statistically significant, assuming that both NMP treatments could be compared to copper hydroxide one in terms of disease reduction.

Disease incidence of bacterial speck was assessed at 7 and 14 dpi. In both cases, either NMP were able to drastically reduce the incidence of the disease, in a comparable way to copper hydroxide, used as positive control (*p* < 0.01). At 7 dpi, theses treated with both NMP showed a disease incidence of 3%, while copper hydroxide at field dose 2.3%. Similar trend was recorded at 14 dpi, with value of disease incidence equal to 3, 2.7 and 3.5% for theses treated with CNC-SHA-CH NMP-HA Starch, CH-CNC-St Starch NMP and copper hydroxide, respectively (Table 5).

In vivo results point out the possibility of using proposed NMP as sustainable substitutes of copper hydroxide, due to the comparable obtained effects in terms of reduction of Pst epiphytical survival and symptomatic expression. These results can be explained considering the afore mentioned antimicrobial properties of chitosan. Moreover, the effects of the NMP different formulation have to be considered. The presence of starch in both tested NMP could have promoted CH release and its consequent antibacterial effects on Pst epiphytical bacterial population. The rapid and statistically confirmed effect on reducing bacterial epiphytic survival of NMP with high amylose starch after the inoculation could be due to the presence of the high amylose starch, which already recorded a greater CH release in the in vitro assays. The presence of more water-soluble polymers in high amylose starch could explain the speed in making available the active principle. Many studies showed high amylose starch has improved properties of controlled drug delivery in relation to conventional starch [80].

A better readiness of the compound involves a higher bacterial inhibition on the inoculated Pst population, so as to allow a reduction in subsequent levels of epiphytic bacterial populations. In support of this, although a similar behavior was shown in plants treated with NMP with standard starch, a general higher n° of CFU cm^−2^ was recorded. Symptoms expression on leaves is related to levels of bacterial population – indeed, where lower CFU cm^−2^ are recorded, an improvement of disease indices can be observed, in terms of severity, incidence and proportional symptoms reduction [81,82]. Furthermore, in elucidating effectiveness of proposed NMP, another crucial aspect could be involved, which is related to the chitosan property as a plant resistance elicitor. Several resistance induction mechanisms are attributed to chitosan, such as phytoalexins synthesis and plant pathogenesis-related proteins production stimulation [83]. Chitosan is also involved in the systemic acquired resistance of plants, through the induction of genes related to jasmonic acid pathways and protease inhibitors [84]. It has been stated that chitosan plays an important role in plant elicitation mechanisms, acting through cell membrane depolarization as an elicitor in the early phases of these processes [85]. Resistance elicitor properties mainly depend on chitosan molecular weight, the degree of polymerization and acetylation and oligomers compositions [20]. Foliar application of high-density chitosan in tomato plants affected by bacterial spot revealed an increase of peroxidases, which are associated to lignin deposition and the production of toxic metabolites for pathogens [32]. Further experiments are unfolding in order to study expression of genes related to resistance mechanisms in tomatoes when both seeds and adult plants are treated with chitosan, especially in alternative nanometric forms. Additionally, CNC and starch could also have had a role in diminishing bacterial infections, creating an unsuitable environment for Pst acting as physical barriers around penetrations sites [53]. There is a high probability that in vivo antimicrobial properties of tested NMP could derive from the interaction of all cited effects.

## 4. Conclusions

The present study describes the effects of an innovative green organic nanostructured compound to counteract tomato bacterial speck disease. Obtained results showed how chitosan hydrochloride, a more water-soluble form of chitosan, possesses a full inhibitory activity on *Pseudomonas syringae* pv. *tomato* at low concentrations (0.5% w/v). In order to facilitate chitosan hydrochloride dispersion on plant canopy and to control its release during time, a nanostructured pesticide was synthetized by the spray-drying technique. Cellulose nanocrystals, due to their chemical properties—among which was the already documented biocompatibility—were proposed as nanocarriers. At the same time, two different types of starch, extracted from a standard and a high amylose content bread wheat cultivars, were investigated to assess their effects on chitosan hydrochloride release. Results confirm the possibility of using the Cadenza SBEIIa genotype as source of high amylose content starch. Obtained nanostructured microparticles were characterized for their biochemical properties, revealing a mean size of 2.9–3.8 µm, and an achieved loading of 50% w/w in terms of chitosan hydrochloride content. In vitro chitosan release assay has, as expected, shown a higher release for microparticles embedded with high amylose starch, capable of releasing half of loaded chitosan after 24 h, while microparticles with standard starch reached the 30%. Both of microparticles when used at 1% w/v were able to fully inhibit bacterial growth after 48 h, showing a bacteriostatic effect that persists at 72 h, with a slightly decrease for microparticles with standard starch. In vivo experiments assessed the complete biocompatibility of proposed nanostructured pesticides when foliar is applied at 1% w/v, demonstrating no harmful effects on the vegetative development of tomato plants. At the same time, both microparticles were able to diminish the epiphytical bacterial survival and the disease incidence at seven and fourteen days after artificial Pst inoculation. At seven days after inoculation, a significant drop in disease severity with a proportional symptoms reduction was observed, ranging from 45% to 49%, comparable to the one showed by copper hydroxide. This study highlights the concrete possibility of using an organic nanostructured pesticide in replacement of copper, opening new scenarios in sustainable tomato crop protection strategies against bacterial diseases. Further studies have been programmed in order to better clarify chitosan hydrochloride effects on tomato resistance induction, and to investigate nanostructured microparticles behavior in field, as well as to study, through the application of fluorescent markers, uptake and translocation of proposed nanopesticides in living plant tissues.

## Figures and Tables

**Table 1:** Characterization of starch composition in different bread wheat genotypes. Data are represented as the mean and SD, different letters (a, b) show significantly different values after one-way ANOVA, followed by Tukey’s HSD post hoc test were performed.

Genotype	Total Starch (%)	Resistant Starch (%)	Amylose (%)
Cadenza	58.9 ± 0.1 a	0.8 ± 0.2 a	32.0 ± 0.5 a
Cad-SBEIIa	57 ± 0.9 a	6.9 ± 0.4 b	63.1 ± 2.9 b

**Table 2:** Chitosan content and yield for the spray-dried NMP preparations containing St Starch or HA Starch. Replicates are displayed to highlighted powder content homogeneity (n = 4, n = 3).

CH-CNC-HA Starch NMP						
Sample	Powder (g)	Chitosan (g)	CC (% w/w)		%RSD	Yield
			Replicates	Mean ± SD		
A1	0.0041	0.00176	43.0	45.1 ± 2.9	6.4	52%
A2	0.0042	0.00184	43.9			
A3	0.0045	0.00222	49.3			
A4	0.0045	0.00199	44.2			
**CH-CNC-St Starch NMP**						
**Sample**	**Powder (g)**	**Chitosan (g)**	**CC (% w/w)**		**%RSD**	**Yield**
			**Replicates**	**Mean ± SD**		
B1	0.0039	0.00167	42.8	46.7 ± 3.5	7.5	55%
B2	0.0041	0.00196	47.8			
B3	0.0040	0.00198	49.5			

**Table 3:** Tomato seedlings’ biological parameters measurements for each treatment at different times. Three leaves per plant were collected to assess mean area using a scanning software. Three measurements on the second leaf of each plant were performed using a leaf clip sensor to assess chlorophyll and flavonols content, whose ratio was used to calculate nitrogen balance index. Means and SD are reported after one-way ANOVA was performed.

Treatment	Biological Parameters	Days Post Treatment		
		1 dpt	7 dpt	14 dpt
Water	Leaf Area (cm^2^)	3.8 ± 0.1	4.0 ± 0.2	7.1 ± 0.7
	Chlorophyll Content (DU)	27.6 ± 1.0	26.9 ± 1.9	28.0 ± 0.6
	Flavonols Content (DU)	0.56 ± 0.04	0.39 ± 0.01	0.41 ± 0.02
	Nitrogen Balance Index	59.3 ± 3.5	71.2 ± 5.1	71.4 ± 2.5
Copper Hydroxide	Leaf Area (cm^2^)	3.4 ± 0.1	5.6 ± 0.5	7.8 ± 1.2
	Chlorophyll Content (DU)	27.8 ± 1.3	29.4 ± 1.0	28.2 ± 0.8
	Flavonols Content (DU)	0.74 ± 0.06	0.44 ± 0.03	0.38 ± 0.01
	Nitrogen Balance Index	45.1 ± 4.4	71.2 ± 3.4	75.6 ± 2.3
CH-CNC-Standard starch NMP	Leaf Area (cm^2^)	3.7 ± 0.2	5.0 ± 0.6	7.3 ± 0.8
	Chlorophyll Content (DU)	26.2 ± 1.1	26.1 ± 0.6	28.6 ± 1.0
	Flavonols Content (DU)	0.58 ± 0.04	0.45 ± 0.03	0.38 ± 0.01
	Nitrogen Balance Index	50.9 ± 3.6	63.0 ± 2.9	75.8 ± 2.8
CH -CNC-High amylose NMP	Leaf Area (cm^2^)	3.3 ± 0.1	4.9 ± 0.7	7.7 ± 0.7
	Chlorophyll Content (DU)	27.6 ± 0.9	28.1 ± 1.6	28.7 ± 0.5
	Flavonols Content (DU)	0.69 ± 0.06	0.42 ± 0.03	0.46 ± 0.03
	Nitrogen Balance Index	46.2 ± 3.0	69.4 ± 2.9	67.1 ± 3.4

**Table 4:** Disease severity (DS) is expressed as the number of point-like necrosis per plant at 7 dpi. Disease symptoms reduction (DR) was calculated as percentage ratio between plant disease severity, using water as a control. Data are represented as the mean and SD; different letters (a, b) show significantly different values after one-way ANOVA, followed by Tukey’s HSD *post hoc* test were performed.

Treatment	7 Days Post Inoculation *(p* < 0.01)	
	DS (Necrosis Per Plant)	DR (%)
Water	38.1 ± 4.9 a	-
Copper Hydroxide	25.1 ± 2.9 ab	34.1 ± 7.9
CH-CNC-HA Starch NMP	20.6 ± 2.8 b	49.9 ± 7.5
CH-CNC-St Starch NMP	19.1 ± 3.7 b	45.9 ± 9.6

**Table 5:** Disease incidence (DI) of theses calculated as percentage ratio between total necrotized area and total area of collected leaves for the assessing of Pst epiphytical survival. Data are represented as the mean and SD, different letters (a, b) show significantly different values after one-way ANOVA, followed by Tukey’s HSD *post hoc* test were performed.

Treatment	Disease Incidence (%) (*p* < 0.01)	
	7 dpi	14 dpi
Water	4.8 ± 0.2 a	4.9 ± 0.3 a
Copper Hydroxide	2.3 ± 0.2 b	3.5 ± 0.4 b
CH-CNC-HA Starch NMP	3 ± 0.4 b	3 ± 0.2 b
CH-CNC-St Starch NMP	3 ± 0.2 b	2.7 ± 0.04 b
